# Advances in Ultra-High-Resolution Mass Spectrometry for Pharmaceutical Analysis

**DOI:** 10.3390/molecules28052061

**Published:** 2023-02-22

**Authors:** Estelle Deschamps, Valentina Calabrese, Isabelle Schmitz, Marie Hubert-Roux, Denis Castagnos, Carlos Afonso

**Affiliations:** 1 Normandie Univ, COBRA, UMR 6014 and FR 3038, Université de Rouen, INSA de Rouen, CNRS, IRCOF, 1 rue Tesnières, CEDEX, 76821 Mont-Saint-Aignan, France; 2 ORIL Industrie, Servier Group, 13 r Auguste Desgenétais, 76210 Bolbec, France; 3 Université de Lyon, Université Claude Bernard Lyon 1, Institut des Sciences Analytiques, CNRS UMR 5280, 5 Rue de La Doua, F-69100 Villeurbanne, France

**Keywords:** pharmaceutical analysis, ultra-high-resolution mass spectrometry, FTMS, FTICR, Orbitrap

## Abstract

Pharmaceutical analysis refers to an area of analytical chemistry that deals with active compounds either by themselves (drug substance) or when formulated with excipients (drug product). In a less simplistic way, it can be defined as a complex science involving various disciplines, e.g., drug development, pharmacokinetics, drug metabolism, tissue distribution studies, and environmental contamination analyses. As such, the pharmaceutical analysis covers drug development to its impact on health and the environment. Moreover, due to the need for safe and effective medications, the pharmaceutical industry is one of the most heavily regulated sectors of the global economy. For this reason, powerful analytical instrumentation and efficient methods are required. In the last decades, mass spectrometry has been increasingly used in pharmaceutical analysis both for research aims and routine quality controls. Among different instrumental setups, ultra-high-resolution mass spectrometry with Fourier transform instruments, i.e., Fourier transform ion cyclotron resonance (FTICR) and Orbitrap, gives access to valuable molecular information for pharmaceutical analysis. In fact, thanks to their high resolving power, mass accuracy, and dynamic range, reliable molecular formula assignments or trace analysis in complex mixtures can be obtained. This review summarizes the principles of the two main types of Fourier transform mass spectrometers, and it highlights applications, developments, and future perspectives in pharmaceutical analysis.

## 1. Introduction

Pharmaceutical analysis plays a crucial role in the development of safe and efficient drugs by evaluating the adequacy of active compounds either by themselves (drug substance) or in a mixture with excipients (drug products). Different aspects are involved in the assessment and development of medications suitable for human health, e.g., the drug synthesis, its pharmacokinetics and pharmacodynamics, distribution in the target tissue but also bulk drug properties and contamination assessment [1]. Importantly, for their approval and release in the market, medications need to satisfy many requirements imposed by the community standards of a country [2,3] and pass all phases of the clinical trials. For this reason, the pharmaceutical industry results among the most strictly regulated sectors of the global economy. One of the major challenges consists in achieving the opportune balance between rigorous drug testing and the time needed for the approval of efficient and riskless medicines. While assays included on monographs and based on classic techniques such as chromatography, titrimetric analysis, spectrophotometry, and potentiometry are still used as principal methods for routine bulk drug analysis before and during commercialization, research in the field of pharmaceutical analysis is pushing toward the use of cutting-edge and high throughput analytical techniques or untraditional methods for a more comprehensive understanding of a drug included its bulk characteristic and its interaction within a biological system. Importantly, specific analytical methods giving more confident outputs and simplifying long and tedious protocols are required for better drug quality evaluations. Thanks to the ability to perform both qualitative and quantitative analyses and to detect compounds with high sensitivity and specificity up to the trace level, mass spectrometry (MS) represents a gold standard for both routine quality controls and research aims. In the last case, MS has contributed to different fields of pharmaceutical analysis, from small molecules to pharmaceutical biomolecules (e.g., peptides, proteins, DNA), up to highly complex systems of pharmaceutical interests (e.g., intact viruses and bacteria). The ‘chameleonic’ versatility of mass spectrometry is associated with the high number of possible combinations regarding different ion sources, analyzers, and detector setups and the possibility of coupling it with different separation techniques, the two most known being gas chromatography (GC) and liquid chromatography (LC). As ions are separated and detected according to their *m/z* values, respectively, in the mass analyzer and the detector, the construction and functioning of these parts strongly impact the performances of a mass spectrometer. Resolving power (RP, or mass resolution) refers to a mass analyzer’s ability to distinguish between two peaks of slightly different *m/z* ratios (isobars), which correspond formally to ions with different molecular formulas but very close *m/z* (i.e., the same nominal mass). RP can be calculated as (*m/z*)/Δ*_m/z_*, with Δ*_m/z_*measured at the full-width half maximum (FWHM) of the *m/z* peak. According to RP, mass spectrometers have been distinguished [4] in (i) low resolution at RP < 10,000 (e.g., quadrupole or linear ion trap), (ii) high resolution (HRMS) at RP > 10,000 (e.g., time-of-flight (ToF) analyzers) and (iii) ultra-high resolution (UHRMS), for instruments at RP > 100,000, which include mostly Fourier transform ion cyclotron resonance mass spectrometers (FTICR MS), Orbitrap, and some recent ToF instruments. On another side, the accuracy of a mass spectrometer refers to the degree of conformity between the measured *m/z* and the theoretical *m/z*, calculated from a proposed molecular formula [5]. Improvements in the measured resolution and mass accuracy are nowadays a dominant requirement in analytical sciences, especially for the chemical characterization of complex samples. In the last years, HRMS has shown great potential in many applications for pharmaceutical analysis [5,6,7]. On another side, UHRMS instruments offer incomparable resolving power and mass accuracy both for low- and high-intensity ions thanks to their high dynamic range. They may represent a real shift in the use of mass spectrometry for the evaluation of pharmaceuticals, allowing accurate mass measurements (at the order of ppb) and access to the isotopic fine structures. This can lead to the detection and the unambiguous assignment of molecular formulae for thousands of ions in highly complex matrices [8,9]. This review focuses on the applications, limits, and perspectives of ultra-high-resolution mass spectrometry (i.e., Orbitrap and FTICR MS) in pharmaceutical analysis. In the first part of this review, we provide a summary of their functioning and perform a comparison to provide insight into their specific performances and highlight the advantages of the usage of both instrument setups in the pharmaceutical industry. Then, applications and developments of UHRMS in the main fields of the pharmaceutical industry are presented.

## 2. Principles and Comparison of Orbitrap and FTICR Analyzers

### 2.1. Orbitrap Technology

The Orbitrap analyzer was invented and patented by Alexander A. Makarov in 1999. Its principles have been extensively described in the literature [10,11,12]. Briefly, Orbitrap analyzers are constituted of two outer electrodes forming a barrel surrounding a central spindle-like electrode [10]. By applying a DC voltage between the inner and outer electrodes, a quadro-logarithmic electric field is produced. While maintaining the outer electrode voltage, the decrease in the central electrode voltage allows an increase in electric field strength and therefore an ‘electrodynamic squeezing’, which can trap ions of different *m/z* values. Thanks to the C-trap, ions are then injected tangentially to the z-axis offset z = 0 by acceleration at high voltage (in kV). The high tangential velocity generates a centrifugal force that, in conjunction with the electrostatic attraction towards the central electrode, induces the ions to orbit around the central electrode. Moreover, the geometry of the inner and outer electrodes induces harmonic axial oscillations of the ions throughout the path into the Orbitrap analyzer [12]. The axial frequency of an ion (ω) is independent of the initial velocity and position and depends solely on the *m/z* according to the following equation (Equation (1)):

(1) $$\omega =\sqrt{\frac{k}{m/z}\;}$$

where *m* is the mass of the ion, *z* is the charge of the ion, and *k* is the field curvature [10].

To access the axial frequency, the outer electrodes detect the image current created by the axial oscillations of the ions. After amplification and analog-to-digital conversion, the produced time-domain transient is Fourier-transformed to the frequency domain. Then, *m/z* can be obtained using external calibration with standards. Under these circumstances, the mass resolution of an Orbitrap is given by Equation (2):

(2) $$\frac{m}{\Delta_{m50\%}}=C\;\times T_{acq}\times \sqrt{\frac{1}{m/z}}$$

where Δ_m50%_ is the full width of a spectral peak at half-maximum peak height in mass, *C* is a constant, and *T*_acq_ is the acquisition time [12]. Equation (2) shows that higher acquisition times (or transients) lead to higher mass resolution. By convention, Orbitrap mass resolution is given at *m/z* 200. For most Orbitrap instruments, ultra-high resolution can be achieved with transients longer than 1 s. As an example, using the LTQ Orbitrap, a transient of 1536 ms is required for a resolution power of 120,000 FWHM [13].

When coupling LC to MS, the MS analyzer must be fast enough to acquire sufficient data points across narrow chromatographic peaks. On another side, high-resolution values are achieved by increasing the acquisition time. This means that in the case of Orbitrap analyzers, an acceptable LC resolution may lead to lower resolution. Most studies using LC-Orbitrap restrain the resolution power to 60,000–70,000 FWHM as a compromise between LC resolution and mass resolution. Please note that those studies are out of the scope of this review. Nowadays, the development of high-field compact Orbitrap analyzers and improved FT signal processing (eFT) have led to the release of new instruments, e.g., the Orbitrap Elite^TM^ and Orbitrap Exploris^TM^ (ThermoFisher, St, Miami, OK, USA) [14], which can achieve a two- to four-fold resolution increase for the same time transient. As an example, using the Orbitrap Elite^TM^, a transient of 384 ms is required for a resolution power of 120,000 FWHM [13]. Therefore, when coupling these instruments to LC, it is possible to perform UHRMS experiments while maintaining good LC sampling.

### 2.2. FTICR Technology

The first FTICR MS experiment was performed in 1973 by Melvin B. Comisarow and Alan G. Marshall based on an analogy to FT-NMR [15]. The physical principles of the FTICR analyzer have been extensively described elsewhere [8,16,17,18]. A brief explanation will be given below. The FTICR analyzer is an ion trap made of excitation electrodes, detection electrodes, and end-trapping electrodes, surrounded by a high magnetic field magnet. Ions are radially confined by the magnetic field while being axially confined by the end-trapping electrodes. After injection into the ICR cell, ions are excited using radio frequency voltages in order to obtain a detectable coherent ion motion. Due to the magnetic field, ions undergo a circular motion called cyclotron rotation, for which the angular velocity ω_c_ can be calculated using Equation (3) [16]:

(3) $$\omega_{c}=\frac{B}{\frac{m}{q}}$$

where B is the magnetic field strength in T, *m* is the mass in kg, and *q* is the charge in C. The image current created by the ion packets passing near the detection electrodes is then amplified and analog-to-digital converted. The produced time-domain transient is Fourier-transformed to the corresponding frequency domain. Then, *m/z* can be obtained using external calibration with standards. The mass resolution of an FTICR is given by Equation (4):

(4) $$\frac{m}{\Delta_{m}}=\frac{C\;\times B\times T_{acq}}{m/z}$$

where Δ_m50%_ is the full width of a spectral peak at half-maximum peak height in mass, C is a constant, and T_acq_ is the acquisition time [19]. Equation (4) shows that higher acquisition times (or transients) and higher magnetic field strength lead to higher mass resolution. FTICR can confine ions for a longer time than other mass spectrometry analyzers, leading to unbeatable mass resolution [20]. For example, Nikolaev et al. [21] used a 7 T FTICR with a dynamically harmonized cell for the single isotope detection of reserpine (*m/z* 609.28066). A 180 s transient could be performed, leading to a resolving power of 24,000,000 FWHM. However, in most studies, much shorter transient times (i.e., data point size) are sufficient. Using 8 million points, a resolving power of more than 1,000,000 at *m/z* 400 can be achieved with a transient of 12 s or 3.8 s, respectively, for 9.7 T [22] and 15 T [23] instruments. As shown here, since the FTICR is a “slow” analyzer, the coupling to chromatography techniques requires reducing the transient hence the resolving power. To take full advantage of the FTICR resolving power, most studies are performed by direct infusion of the sample. Recently, instrumental optimizations led to the development of the dynamically harmonized FTICR cell, i.e., paracell [17], that allows higher resolution but requires fine-tuning by an expert-hand or experimental design [24]. Moreover, the recent introduction of quadratic detection with the 2ω mode (or 2xR system) allows acquisition and detection on the same electronic circuits and a resolution comparable to FTICR with a 2-times higher magnetic field (e.g., 7 T with 2ω and 15 T with 1ω) [23].

### 2.3. Comparison of the Two Techniques

Although Orbitrap and FTICR mass spectrometers share similarities (e.g., the proportionality of the transient length to the resolution, current image detection, and FT conversion), several differences can be highlighted. The comparison of the two analyzers is delicate since numerous instrument models exist with various technological improvements performed over the years. A global comparison of the two analyzers is given in Table 1.

Orbitraps are popular instruments in the pharmaceutical analysis [6]. They are compact and robust, often sold with LC or GC-coupling setups, have a user-friendly interface, and provide (ultra) high-mass resolution and high-mass accuracy. FTICR instruments provide unbeatable performances in resolution, mass accuracy, and dynamic range. However, they remain costly and need high expertise and an adapted laboratory layout (because of their large size and weight, high magnetic field magnet, and the need for cryogenic maintenance) [25].

For the scope of this review, the discussion is focused on the applications of FT-based analyzers in pharmaceutical analysis, while advances in technical aspects can be found in already published literature [20,26]. In regards to the dynamic range limitations observed in both analyzers due to the coalescence phenomena (merging of close *m/z* ions at high concentration), a discussion can be found for both FTICR [27] and the Orbitrap [28] analyzers.

### 2.4. Activation Methods Using FTICR and Orbitrap

In addition to ultra-high resolution and high mass accuracy, FTICR and Orbitrap mass spectrometers can perform ion activation experiments for the structural analysis of compounds, from small drug-like molecules to intact proteins. The most used techniques are briefly presented here. Some fragmentation methods can be performed in both instruments.

Collision-induced dissociation (CID), also known as collisionally activated dissociation (CAD), uses a neutral gas (e.g., argon, nitrogen) to collide with ions. The conversion of the collision energy to the ions’ internal energy leads to the breaking of the weakest chemical bonds. CID is substantially used for small and large molecules, while its use for proteins with post-translational modifications is limited since information is lost during the fragmentation process [29]. Electron capture dissociation (ECD) involves the introduction of low-energy electrons to multiply charged cations. Then, fragmentation occurs from cleavage reactions of the radical cations [30]. Similarly to ECD, electron-transfer dissociation (ETD) uses anions as electron donors to fragment multiply charged peptide cations. ECD and ETD are particularly useful for protein studies, e.g., top-down sequencing and post-translational modifications (PTMs) analysis [29]. Infrared multiphoton dissociation (IRMPD) activates trapped ions using a low-power (<100 W) continuous-wave (10.6 µm) CO_2_ laser for from ten to hundreds of milliseconds. Fragmentation occurs by the absorption of IR radiation, followed by an internal energy distribution similar to CID [31]. Ultraviolet photodissociation (UVPD) is an ion-activation/ dissociation method using high-energy UV photons. Lastly, in UV photo-absorption, the excitation of ions to excited electronic states enables higher energy-dissociation pathways that are usually not observed upon low-energy collisional activation [32].

While the aforementioned methods are feasible with both Orbitrap and FTICR analyzers, other types of activation methods have been developed specifically on one determined experimental set-up by taking advantage of the peculiar characteristics of the instruments. For example, two-dimensional Fourier transform ion cyclotron resonance mass spectrometry (2D FTICR MS) is a tandem mass spectrometry technique enabling the correlation between precursor and fragment ions without previous precursor *m/z* isolation [33]. This method is based on different steps consisting of (i) excitation of ions for parent *m/z* detection, (ii) de-excitation and ion squeezing at the center of the cell, (iii) fragmentation, and (iv) fragment ion *m/z* detection. Since the presence of gas in the ICR cell destroys the ion packet coherence, CID cannot be used with 2D FTICR MS, and the two most used activation methods are IRMPD and ECD. The obtained 2D mass spectrum presents *m/z* for precursor ions on the y-axis and *m/z* for fragment ions on the x-axis. Another dissociative technique unique to FTICR MS instruments is the electron detachment dissociation (EDD) method, in which backbone cleavage of anions is reached through the collision with medium energy electrons via the creation of a radical site [34]. Another ion activation technique commonly performed on FTICR MS instruments is sustained off-resonance irradiation collision-induced dissociation (SORI-CID). It consists in submitting ions into the FTICR cell to subsequent acceleration-deceleration cycles, which eventually cause multiple collisions at low, transitional energy (<10 eV). Under these circumstances, fragmentation results are softer than classic CID, as increments in ions’ internal energy are smaller [35]. Blackbody infrared radiative dissociation (BIRD) enables ion dissociation reaction at virtual zero pressure induced by the presence of an ambient blackbody radiation field (thermal electromagnetic radiation). Measurements of BIRD rates in the function of the temperature give access to the quantitative observation of thermodynamics parameters, for example, binding and dissociation constants for gas-phase complexes [31,36]. Applications and advantages of these activation techniques for the pharmaceutical analysis of different systems will be gradually presented in the different sections of this article.

Regarding Orbitrap analyzers, higher-energy collisional dissociation (HCD) experiments performed in the C-Trap or the octopole use a neutral gas (mostly nitrogen) with higher energy than in CID with a quadrupole ion trap [37]. Electron transfer/higher-energy collisional dissociation (EThcD) is a fragmentation experiment in two activation steps that can be performed by hybrid-Orbitraps. In EThcD, precursors are accumulated in the linear trap and subjected to electron transfer dissociation. Fragments and unaffected precursors are then subjected to higher-energy collisional dissociation. This fragmentation combination allows better peptide sequencing than HCD alone [38].

### 2.5. Considerations about Sample Preparation for Orbitrap and FTICR-MS Analyses

Sample preparation is crucial to obtain reliable and efficient results based on the use of mass spectrometry techniques, especially in the case of pharmaceutical forms in which compounds of interest are dispersed in complex matrices containing abundant excipients of different natures. Routine practices for MS analysis consist of the extraction of compounds of interest at a sufficient concentration for their detection; and, at the same time, the elimination of the interferences that could bias the analyses, such as salts, macromolecules, and contaminants. Of note, in comparison with the direct introduction, the addition of a separation technique, such as liquid chromatography, allows using simpler clean-up procedures since interferences can be eluted from compounds of interest, e.g., salts elute at the hold-up time in reverse phase chromatography. Moreover, ultra-high resolution leads to higher high peak capacity in the mass dimension; therefore, the peak capacity in the chromatographic dimension can be reduced while maintaining the global method resolution.

Nevertheless, in direct introduction workflows, which involve principally the use of FTICR mass spectrometers with an ESI source, sample preparation must be meticulously optimized. In fact, the ultimate limitation of FTICR MS in direct introduction mode lies in the presence of matrix effects, i.e., ion competition and ion suppression, which are harshened by the very high sensitivity of the instrument [39,40]. While the first is caused by the presence of multiple ions in the source competing as carriers for the charge, ion suppression may be caused by the high abundance ions that lead to reduced sensitivity of the low abundance ones. Biases may arise in the analysis of minor pharmaceutical compounds in the presence of abundant excipients or in samples containing a residual salt concentration. Analyte extractions from complex matrices and contaminant removal can be achieved with classic separation methods, such as solid phase extraction (SPE), liquid-liquid extraction (LLE), or precipitation by the addition of immiscible solvents to the starting solution. In particular, SPE based on the use of non- or mid-polar sorbents [41,42] or magnetic beads [43] represents the preferred technique to produce FTICR MS compatible samples, offering both small compounds extraction and desalting, even if partial loss of (polar) molecules is often experienced. In addition, dialysis and electrophoresis represent the preferred techniques for the isolation and purification of protein-derived material.

In the alternative, when sample preparation optimization is not possible, the use of other ionization sources, for example, MALDI, Atmospheric Pressure Chemical Ionization (APCI), or Atmospheric Pressure Photoionization (APPI), may help in analyzing complex mixtures with reduced matrix effects [44].

Finally, for MS imaging applications covering the field of pharmaceutical research (Section 5), thin and flat surfaces are required for the homogeneous ionization of compounds of interest. Human or animal tissues are normally cryo-sectioned on a microtome or treated according to the sample stretching method [45].

A thorough evaluation of sample preparation methods for pharmaceutically active compounds compatible with MS analysis has been recently reviewed by Almeida [46].

## 3. Ultra-High-Resolution Mass Spectrometry for Drug Discovery and Structural Characterization

Drug discovery consists in identifying and developing new drugs for the treatment of specific diseases and medical conditions. This process typically involves several steps, including target identification, lead compound screening, and preclinical and clinical testing. Classic drug discovery approaches are based on high-throughput screening (HTS), fragment-based drug discovery (FBDD), structure-based drug design (SBDD), or ligand-based drug design (LBDD) [47]. The obtained “lead” compounds are then optimized in terms of their potency, selectivity, physicochemical properties, and pharmacokinetic and toxicity properties [48]. Another strategy for drug discovery consists in isolating bioactive compounds from a complex mixture of vegetal or animal origin [49,50]. In fact, natural products are a rich source of medicinal compounds with intrinsic pharmaceutical activities.

On another side, structural characterization refers to the determination of the detailed atom connectivity, isomerism, and three-dimensional structure of a drug molecule, which can help to understand its function and design more effective drugs. In this regard, the characterization of therapeutics is of utmost importance in the pharmaceutical industry for the production and release of efficient and non-toxic drugs. Knowledge of molecular structure can help scientists understand pharmaceutical effects, imagine new and more performant related drugs, and foresee chemical modification which could lead to toxic secondary compounds and, eventually, releases efficient and harmful medicines to commerce. Structural characterization is typically performed using techniques such as X-ray crystallography, nuclear magnetic resonance spectroscopy, or computer modeling [51,52]. Nevertheless, thanks to the multitude of existing analyzer geometries and experimental settings, mass spectrometry is becoming a technique of choice not only for quantitative analysis but also for performing structural analysis. In today’s competitive landscape, modern drug discovery puts emphasis on speed and high throughput methods because of the necessity to satisfy the release of new and more efficient medicine into the global market and find new therapies for rare diseases or pathological conditions that do not still have one. Therefore, ultra-high-resolution mass spectrometry results in a valid ally enabling access to a high number of molecular information on thousands of molecules in a few minute runs, avoiding long extractions and purification of samples. Orbitrap and FTICR-based mass spectrometers are increasingly used in the pharmaceutical industry for the possibility of measuring accurate mass-to-charge ratios, which can often enable a direct interpretation of the studied system without acquiring additional information, as in the case of low-resolution mass spectrometers. Moreover, in addition to accurate measurement, high-resolution mass spectrometry can produce compositional and structural information on small molecules, biomolecules, and complexes. In the next sections, we will present recent articles on the use of these MS-based techniques for the discovery and structural characterization of very diverse active compounds.

### 3.1. Small Molecules Drugs

Small molecules are chemical compounds with a molecular weight typically below 1200 Da. Low molecular weight drugs can enter easily into the cells and bind to specific targets, such as proteins or enzymes, to produce a desired therapeutic effect. They are commonly used in the pharmaceutical industry to treat a variety of medical conditions. In the last decades, the advantages of the use of ultra-high-resolution mass spectrometry have been especially exploited to perform global profiling and discovery of new compounds in natural complex mixtures, either already used in traditional and alternative medicine or constituting new unexplored sources of active compounds. For example, humic substances (HS) are produced during the decomposition of decaying biomass and contain a large variety of compounds, e.g., lignins, lipids, tannins, and carbohydrates. Despite their recognized antiviral, antibacterial, and anti-inflammatory activity, not all compounds responsible for the pharmacological effect have been identified. Thanks to the ultra-high resolving power of an FTICR-based mass spectrometer, Orlov et al. [53] assigned over 6500 different compounds in eight natural HS samples, discovering the existence of 100 common species, which were further investigated for the discovery of potential compounds with pharmacological activity. Thanks to the unique molecular formula obtained for each compound, the authors linked the pharmacological activity to the presence of flavonoid-like structures. Then, by crossing compound structural information obtained from selective isotopic exchange and formulae searching in the ChEMBL database, the authors could restrain the results to a set of 49 compounds responsible for the biological activity. Similarly, FTICR MS, coupled with ESI and paper spray ionization (PSI), has enabled the fast and accurate identification of 28 psychoactive substances in illegally seized blotterpaper and tablets without the need for complex sample extraction. In the same article, fine isotopic structure and CID spectra obtained in ultra-high resolution enabled unequivocal formula assignment and structure elucidation of psychoactive drugs containing single or multiple atoms of chlorine and nitrogen [54]. A similar approach has been used for the characterization of highly complex mixtures with pharmacological activity as herbal fermented beverages [55], synthetic cannabinoids [56], or for the identification of new active ingredients with anticancer activity in the *Euphorbia tirucalli* latex [57]. It is worth noting that accurate fragmentation mass spectra obtained by FTICR are very useful in drug discovery as they result in very highly informative data for the screening of possible targets. Thanks to ultra-high-resolution fragmentation spectra and database screening, Yang et al. [58] found that arborside E, an active compound in the extract of *Psydrax montigena,* possesses binding activity for malaria targets. In a similar way, Vu et al. [59] found 96 low-molecular-weight natural products identified as binding partners of 32 of the putative malarial targets.

While an increase in resolving power allows discrimination among isobars, allowing rapid global profiling of complex mixtures even in direct injection mode, it still does not ameliorate the analysis of structural isomers which have the same *m/z* ratio. As it happens in other fields, separation, and identification of small isomeric compounds of pharmaceutical interest can be achieved through coupling with chromatography techniques. As stated before, this coupling can lead to a decrease in the mass resolution of Orbitrap and FTICR mass spectrometers. Nonetheless, these assets remain advantageous for the analysis of small molecular weight pharmaceuticals with respect to classic high-resolution mass spectrometry, regarding (i) accurate mass measurements for both MS1 and MS2 levels and (ii) access to a fine isotopic structure that enables more confident molecular formula assignments. For example, isomeric sulfur-containing metabolites with health benefits in *Allium* vegetables have been characterized through LC-FTICR MS and LC-FTICR MS/MS. In this work, authors highlighted that ultra-high-resolution fragmentation spectra obtained in communion with isomeric separation through liquid chromatography allowed the discrimination of γ-glutamyl-S-1-propenylcysteine (Compound 2) and γ-glutamyl-S-2-propenylcysteine [60]. The literature presents a very high number of studies performed via LC-ESI-FTICR for the study of natural products in complex mixtures with biological activity [61,62,63], in which annotation of compounds and fragments has been obtained with mass precision lower than 1 ppm. Moreover, ultra-high-resolution mass spectrometry shows very high potential in the discovery and characterization of N- and S- containing molecules, which are of high interest in the pharmaceutical industry thanks to their known biological activities. While the presence of a naturally abundant ^34^S isotopic ion is a common structural feature of S-metabolites, in the case of N-containing metabolites, unambiguous formula assignment of N- containing or NS-containing active molecules can be obtained by measuring mass shifts of peaks corresponding to labeled (^15^N) and non-labeled (^14^N) compounds, denoting the number of N atoms. These approaches based on the comparison of fine isotopic structures of compounds containing stable or labeled isotopes has been applied in the discovery of new compounds with anticancer activity in *Catharanthus roseus* [60] or for the fast and efficient target identification of S-compounds with antihypertensive properties in the very complex matrix of *Asparagus officinalis* in which over 4537 peaks have been detected [64].

### 3.2. Proteins and Antibodies

In the last decades, the biopharmaceutical industry has placed a significant emphasis on the research and development of protein-based pharmaceuticals. Thanks to their efficiency and broad applications, proteins are becoming one of the fastest-growing therapeutical strategies to cure or prevent important pathologies. To cite some examples, the antidiabetic drug insulin is a peptide hormone, anti-SARS-CoV-2 infection therapies are based on the use of monoclonal antibodies, and modern vaccines are derived or based on bacterial and virus peptides. Nevertheless, compared to conventional small molecule drugs, the discovery and analysis of protein-based pharmaceuticals may be hindered by their size and structural variability. Importantly, both the functionality and safety of protein-based drugs depend not only on the primary structure but also on their complex tridimensional conformation (secondary, tertiary, or quaternary structure). Therefore, the structural analysis of proteins needs highly through-put analytical techniques to deconvolute their intrinsic structural heterogeneity. Historically, protein identification has been based on bottom-up experiments consisting in the enzymatic digestion of peptides (up to 4 kDa) that are then analyzed through LC-MS/MS based on high-resolution mass spectrometry. Sequence identification is then obtained by comparison of experimental and in silico peptide fragmentation spectra (obtained from genomics or transcriptomics data). The introduction of soft ionization techniques (principally ESI or MALDI) has enabled the analysis of intact proteins (native MS or top-down with MS/MS), allowing a complete investigation of structural properties of peptides, including post-translational modification (PTMs) [65]. Top-down proteomics gives information regarding the different proteoforms (e.g., the same protein backbone with different PTMs), allowing the determination of the peptide’s primary structure and possible modifications [66]. However, the analysis of large proteoforms and proteins characterized by multiple coexisting PTMs is technically challenging due to the high molecular weight of the studied compounds and the formation of complex spectra, which are difficult interpretation [67]. In the last years, middle-down MS has emerged as a compromise between bottom-up and top-down experiments. In this approach, proteins are lysed to large peptides (i.e., polypeptides, up to 12 kDa [68]) with more coexisting PTMs than the peptides obtained by the bottom approach [67].

Independently from the type of approach, mass spectrometric characterization of proteins requires the specificity afforded by ultra-high-resolution mass measurements performed at both the intact mass and product ion levels. They provide accurate mass detection at high *m/z*, separation of isobaric proteins in complex mixtures, enable access to the fine isotopic structure for highly charged ions, and allow diverse fragmentation modes. Importantly, with respect to time-of-flight (ToF) mass spectrometers, FTICR mass spectrometers can achieve much higher mass resolution (higher than 1 M with respect to 100,000 for ToFs), access to the theoretical unlimited mass range and the possibility to manipulate the ions in the gas phase. As opposed to ToF-MS detection, detection in the ICR cell is non-destructive, and it enables gas-phase ions to keep their original conformation for longer periods. For these reasons, FT-based instruments are increasingly used for the analysis of kilo-or megadaltons proteins through native and top-down approaches [69]. Nevertheless, it is worth noting that because of their easiness and contained cost, Orbitrap analyzers remain the preferred platforms for the analysis of proteins and proteoforms < 50 kDa, while FTICR-based mass spectrometers are used in native approaches for proteins and antibodies at molecular weight >100 kDa [70]. To the best of our knowledge, the heaviest proteins which have been detected and characterized correspond to 16-mers β-galactosidases at 1.8 MDa, obtained through a 15 T FTICR MS platform [71].

Regarding top-down applications, it is worth noting that different activation methods may be necessary to obtain a full understanding of the studied system and that they turn out to be complementary [72]. For example, ISD, CID, and IRMPD cause fragmentation of the protein backbone at the amide bonds generating b/y-type of fragment ions, while ECD cause principally cleavage at the N-Cα bonds and yields c′/z^•^ ions [66]. Importantly, in ECD (and ETD), the fragmentation happens before vibrational energy distribution, leading first to the fragmentation of the protein covalent backbone while retaining non-covalent and labile interactions as the ones responsible for PTMs [30]. This specific ability makes ECD a very useful tool for the characterization of native proteins and protein complexes, allowing it to localize N- and C-terminal subunits in relation to protein complex structure. Of note, top-down ECD approaches based on FTICR MS differ from native top-down MS based on CID in modified Orbitrap instruments because they allow keeping native structures after ejection of a subunit, enabling to obtain information on proteins’ quaternary structure [71].

In pharmaceutical applications, ultra-high-resolution mass spectrometry has been extensively used in the characterization of antibodies, antibody–antigens interaction, and in the characterization of proteins involved in important diseases. For example, native MS and middle-down de novo sequencing through the integration of different FT-based platforms (21 T FTICR, Q Exactive HF^TM^, Orbitrap Fusion Lumos^TM^, and Orbitrap Eclipse^TM^) were used to obtain an in-depth characterization of non-covalently associated hetero-tetrameric immunoglobulin [73]. Paris et al. [74] used 2D-FTICR for the analysis of the post-translational modifications (PTM) of the immunoglobin IgG1 with a bottom-up approach. For this, the antibody and a modified variant were compared. Discrepancies between the two 2D-mass spectra showed the PTM localizations. Thanks to the resolution of the FTICR analyzer, distinct MS/MS spectra of precursors with less than 0.1 Da apart could be obtained. As a comparison, the quadrupole, one of the most used analyzers for MS/MS isolation, offers a mass window from one to several Da. More recently, UVPD has been implemented and optimized as a fragmentation method for 2D-FTICR MS in the data-independent acquisition and coupled to IRMPD activation post UVPD to double the overall fragmentation yield and improve the top-down characterization of ubiquitin [75]. In another case, Jin et al. [76] used LC-Q-Exactive-MS for the verification of monoclonal antibody therapeutics using top-down, middle-up, and bottom-up methodologies on three reference materials and one therapeutic product. UHRMS was used to acquire the peptide sequencing data for the bottom-up experiments. Authors demonstrated that when compared with sequences from the literature, discrepancies and charge variants were localized to the subunit and subdomain levels. Then, these results were used to strengthen the formula assignment of the intact proteins. Their methodology allowed the differentiation and verification of therapeutic antibodies from reference antibodies, whereas the sequence information was not yet publicly available. Similar approaches have been used in pharmaceutical research for the characterization of Bruton’s tyrosine kinase (BTK) in cancerogenic B-cells [77] or to monitor glycation levels at subunit levels in monoclonal antibodies [78]. Larson et al. [79] developed a methodology using LC-ESI-FTICR-CID to analyze reduced (lysis of disulfide bonds) antibody-drug conjugates. This method allowed the determination of the drug-to-antibody ratio, detection of drug variants, and characterization of drug conjugation. Watts et al. [80] developed a method to characterize di-selenide bridging patterns of synthetic seleno-proteins by a dual top-down and bottom-up approach using LC-ESI-Orbitrap-MS/MS (120,000 FWHM). The activation of ions was performed using UVPD and EThcD, which allowed the complete characterization of seleno-protein constructs. In another study, surface-induced dissociation (SID) under high mass accuracy and ultra-high resolution offered by FTICR unveiled that the bacterial enzyme, manganese oxidase, bound to a variable number of copper ions, which were previously thought to be iron ions according to previous lower resolution spectra [81]. Similar approaches were used for the characterization of iron-sulfur proteins [82], the interaction between β-amyloid and metal cations (Cu^2+^, Co^2+^, Ni^2+^, Fe^3+^, and others) in native top-down proteomics [83], and the elucidation of metal-mediated assembly pathway of encapsulated ferritins proteins [84]. Finally, the direct ESI-FTICR-MS assay applied to complexes formed by human serum albumin (HSA) and different flavonoids has allowed the quantification of binding constants and their dependence on flavonoid structure based on comparison of relative peak intensity ratios of HSA–flavonoid complexes and free HSA in the ESI-FTICR mass spectra [85]. Other applications for the use of FTMS (FTICR or Orbitrap) for the top-down sequencing of non-tryptic peptides have been extensively reviewed by Lebedev et al. [49]. Of note, interpretation and characterization of proteins and antibodies through FT-based mass spectra may be harshened by the occurrence of artifacts, i.e., unexpected peaks which differ from those obtained by linear processing of information encrypted on the isotopic fine structure level. These artifacts may arise from transient apodization prior to FT processing in the presence of isotopic envelopes in which closely separated ion signals have nearly equidistant frequencies. In this case, constructive and destructive interference from multiple sinusoidal may change the number of beat patterns of the FTMS transient. Under these circumstances, the application of an apodization function can be deleterious for signal-to-noise and resolution in obtained mass spectra. In silico tools are nowadays commonly used to estimate the appropriate transient length and simulate corresponding beat patterns and resulting FT isotopic distribution spectra [86]. Recently, Nagornov and colleagues [87] released an upgraded and updated FTMS simulator to assess the influence of the charge-state distribution of monoclonal antibodies (mAb) on isotopic beats, including the detection of higher-order harmonics. The authors highlighted the ability of this in silico tool to help in the experimental design and data analysis of Orbitrap and FTICR MS-based instruments extending its application to proteins, viruses, and complex polymers.

While the analysis of large biomolecules would benefit from the ultra-high resolving power and mass accuracy of FT-based analyzers, the low acquisition rate to reach ultra-high resolving power still represents one of the biggest inconveniences pushing scientists to still favor the performance of time-of-flight mass analyzers upon Orbitrap and FTICR mass spectrometers. Improvements in acquisition speed for ultra-high resolving powers can be achieved through frequency multiple detection schemes in FTICR-MS [88]. These assets can be obtained using multi-electrode ion traps (usually called nX-ICR cells), which enable high-resolution mass spectra at proportionally increased acquisition speed without the need to impose changes to the magnetic field strength. In this regard, a 21 T FTICR mass spectrometer coupled to 4X frequency multiplication, ion trapping field harmonization, and spectral data processing methods has recently been applied in the analysis of large intact proteins. In the study, ubiquitin spectra were acquired within a period of 12 ms, and larger biomolecules such as apo-transferrin (MW = 78 kDa) or mAb (MW = 150 kDa) were isotopically resolved in a detection period of 384 and 768 ms [89].

### 3.3. Nucleic Acids and Other Systems of Pharmaceutical Interest

The pioneering work of Ganem et al. [90] on enzyme-substrate and enzyme-product complexes demonstrated that non-covalent associated molecules in solution can be easily transferred into the gas phase through soft-ionization sources (principally ESI and MALDI) and detected as intact complexes. These discoveries have opened new frontiers in drug development as they allow us to obtain insight in a direct way into hot topics in pharmaceutical sciences, e.g., information on ligand-target interactions, stoichiometry, and affinity. In the previous paragraph, we highlighted the capability to use peculiar activation methods for the top-down characterization of protein complexes and PTMs.

In addition to that, direct sample introduction, through a soft ionization source, coupled to ultra-high-resolution tandem mass spectrometry has been extensively used in pharmaceutical analysis to characterize other types of non-covalent complexes (for example, DNA–DNA and RNA–RNA structures) to investigate their interaction with active compounds, or to obtain insight on the inclusion and release of drugs in other systems of pharmaceutical interest. Similarly to proteins, FTICR MS is particularly suited for the analysis of voluminous non-covalent complexes because it gives access, in addition to classic CID experiments, to other fragmentation techniques such as SORI-CID, EDD, IRMPD, and BIRD. These techniques are particularly suited for nucleic acid complexes because (i) non-covalent large ions in the gas phase require multiple collisions and sufficient internal energy for their dissociation, (ii) they allow efficient fragmentation of multiply charged anions (as for example, nucleic acids in gas-phase) which are not responding to EAD and ECD, and (iii) they give access to quantitative characterization of binding and dissociation energies for weak non-covalent complexes and solvated ions. A practical example of the information given by these fragmentation techniques is represented by the study performed by Xu et al. [91], in which the authors studied the noncovalent complexes of DNA with Hoechst 33258, a fluorescent dye used for pharmaceutical applications by ESI–FT/ICR MS in various activation modes. While fragmentation under CID provoked unzipping of the strands, giving only a clue on the localization of the binding site of the drug, IRMPD and SORI–CID experiments mainly gave DNA backbone cleavages and internal fragment ions, allowing a more confident description of the binding site of the drug into the DNA structure. Finally, under EDD fragmentation, no significant dissociation was observed, which led to the conclusion that the drug strongly bound with the DNA, forming strong salt-bridge interactions which stabilized the non-covalent complex. SORI-CID experiments have also been used to characterize different 7-charged flavonoids/DNA complexes, enabling to highlight effects of flavonoid glycosylation and planarity on changing the affinity and the binding modes of the flavonoids in the DNA structure [92]. Thanks to a similar approach, advancements in the development of HIV-1 antiviral strategies have been obtained by studying the inhibitory effect on the normal backbone fragmentation of the ψ-RNA HIV domains, mediated by the non-covalent binding of the aminoglycoside neomycin [92,93].

More recently, Wootton et al. [94] studied the metalation of a DNA 12-mer oligonucleotide by the anticancer complex Os1- Cl·PF6 using ESI-FTICR with CID, IRMPD, and electron detachment dissociation (EDD) fragmentations. The authors showed that CID and IRMPD produced fragments that lost the osmium, whereas EDD allowed the identification of the two binding sites. Top-down MS analysis with collision-induced dissociation (CID) fragmentation has been performed to study mono- and di-platinated single-stranded oligodeoxynucleotides, allowing the unambiguous identification of DNA platination sites towards cytosine and thymine rich- sequences in addition to the more recognized guanine binding sites, and contributing to a better understanding of cisplatin anticancer drugs [95]. Ultra-high-resolution FTICR MS under CID-MS allowed to detect and localize the presence and position of methylation in a natural riboswitch scaffold with self-methylation activity in RNA material. The peculiar activation techniques produced a complete set of c and y RNA fragments allowing complete sequence determination [96]. Finally, ultra-high-resolution mass spectrometry based on IRMPD fragmentation allows studying chiral discrimination in host-guest complexes, as recently demonstrated for L- and D-penicillamine in β-cyclodextrin [97]. Other applications of ultra-high-resolution mass spectrometry for the characterization of complexes of pharmaceutical interest have been recently published in drug encapsulation and drug release from cyclodextrins [98,99,100], micelles [101,102], and nanoparticles [43,103] host-guest complexes.

## 4. Ultra-High-Resolution Mass Spectrometry for Drug Formulation Studies

The pharmaceutically active compound (API) in its final form (drug product) is part of a formulation with excipients. Excipients are substances that have been appropriately evaluated for safety and are intentionally included in a drug product. They can have different functions: drug stability (e.g., light degradation or microbic biodegradation prevention), bioavailability enhancement (e.g., solubility increase, encapsulation), manufacturing reproducibility (e.g., process lubricant), and patient usability and appreciation (e.g., the volume filling for tablets, taste). They may include many physically and chemically diverse compounds as small molecules (e.g., phenols), non-ionic surfactants (e.g., polysorbates), polymers (e.g., polyethylene glycol), nanoparticles, and inorganic compounds (e.g., talc) [104]. The manufacturing and degradation of API and excipients can lead to the presence of impurities, and unwanted chemical substances with no therapeutic benefits or even potential harm to patient safety, if present above a certain limit [105]. Moreover, some impurities can be reactive (e.g., peroxides) and impact product quality and patient safety, even at trace levels [106]. To anticipate possible impurity formation, forced degradation studies are performed on API and drug products by exposition to heat, humidity, and light for solid-state studies and/or to a range of pH values for solution-state studies [107]. Guidelines on impurity characterization in drug products are emitted by the International Council for Harmonisation of Technical Requirements for Pharmaceuticals for Human Use (ICH). According to ICH rules, any impurity whose content is greater than 0.1 % must be identified and characterized. Any potential genotoxic compound needs to be removed by modifying the production process or by reducing its concentration in order to respect the limits of toxicological concern [105]. The assessment of impurities is classically performed through chromatography, even if the advantages of high-resolution mass spectrometry (HRMS) for impurity profiling, quality control, and surveillance have been described in the literature [6], foretelling a possible HRMS implementation in regulated control methods. Impurity studies are numerous, as shown by the number of publications (from 2018 to 2022) on google scholar using the keywords “orbitrap”, “drug”, and “degradation” (17500) or “orbitrap”, “drug”, and “impurity” (1430). However, they are mostly performed with quantitative settings, which enhance sensitivity and/or fast scanning at the expense of resolution. Despite the power of UHRMS to analyze complex mixtures and provide fine isotopic distributions for confident identifications, hardly any of these experiments were performed using UHRMS settings. Even though only a few applications will be described here, the number of publications using UHRMS for formulation analysis might increase in the future. Complex drug excipient compositions impact the performance of the formulation in vivo and consequently affect drug absorption. Hence, differences in excipients from different suppliers or batches can have an impact on the effectiveness of a drug product.

Among different classes of excipients used in the pharmaceutical industry, polymers are currently used to enhance drug delivery by improving the solubility of poorly water-soluble drugs. Polymer analysis is challenging due to their solubility properties, the presence of modifiers that may interfere during the analysis, and the generation of highly complex spectra presenting many isobaric species and multiply charged ions. For this reason, UHRMS and its unrivaled resolving power represent a powerful technique for polymer analyses, especially for the identification and characterization of the different repeating units and terminal groups. Hurtado et al. [108] compared two different batches of Gelucire 44/14 and polysorbate 80 using ESI-FTICR. The two batches of polysorbate 80 were differentiated by four polymeric series, whereas the two batches of Gelucire 44/14 were differentiated by one compound and three polymeric series. Authors highlighted the advantage of ultra-high resolution for the identification of close polymeric distributions, e.g., series of (C_12_H_23_O_2_·[OCH_2_CH_2_]nNa_3_)^3+^ and (C_22_H_42_O_3_·[OCH_2_CH_2_]nNa_3_)^3+^ with a 0.02 Da difference between neighboring peaks. The advantage of ultra-high-resolution mass spectrometry for polymer-based excipients analysis is clearly highlighted in the case of complex derivatized molecules (e.g., poly(ethylene glycol)s functionalized with fatty acid chains), especially when coupled with MALDI ionization, which reduce sample preparation problems by allowing the direct analysis of derivatized-polymer in its crystallized form. For example, Desport et al. [109] analyzed a stearate functionalized poly(ethylene glycol) by MALDI-Orbitrap (240,000 FWHM), identified the repeating unit as C_2_H_4_O and highlighted the presence of six polymeric mass distributions originating from different terminal group combinations. In a similar way, ultra-high-resolution mass spectrometry can help in resolving structured triacylglycerols, a complex mixture of triacylglycerols (TG) with various fatty acid chain lengths. These are used in the pharmaceutical industry for fat emulsion injections, in which their composition and proportion are closely related to the emulsion stability. Hence, their fine characterization is of utmost importance for the release of stabilized injectable medicines. Zheng et al. [110] performed an LC-ESI-FTICR analysis for the identification and relative quantification of forty-seven triglycerides. The identification was performed based on accurate *m/z*, isotopic distribution, MS², and retention times—which are related to the number of carbon atoms and double bonds. The relative quantification results showed that the main triacylglycerol components (>50%) were triacylglycerols containing one medium-chain and two long-chain fatty acids, e.g., TG (8:0/18:2/18:2) and TG (8:0/18:1/18:3).

One of the most advantageous aspects of UHRMS is the access to the fine isotopic distribution of an ion, helping in the confident assignment of molecular formulae through the comparison of experimental and theoretical isotopic distributions. To this scope, access to fine isotopic structure can be achieved at very high resolving power, which can be obtained mostly in direct injection mode. For example, Li et al. [111] analyzed through LC-ESI-FTICR MS capsules containing Cefetamet pivoxil hydrochloride, in which thirteen impurities were identified. In these first measurements, authors observed that even with a mass accuracy of less than 1 ppm, several molecular formulae could be assigned to each exact mass. Better results were obtained after collecting different fractions, which were then analyzed by direct infusion-ESI-FTICR. Thanks to the ultra-high resolution of the FTICR instrument, peaks due to the contribution of ^34^S, ^15^N, ^13^C (Δm^34^S-^15^N^13^C = 0.458 mDa), ^18^O (Δm^15^N^13^C-^18^O = 0.386 mDa), and ^13^C_2_ (Δm^15^N^13^C-^18^O = 0.263 mDa) could be separated. Isotopic fine structures and unique molecular formula assignments can then be combined with MS/MS experiments to perform structural elucidations. In a following study, impurities of Cefetamet pivoxil obtained under different conditions (i.e., hydrolytic degradation (acidic and basic), oxidation, photolysis, and thermal degradation) were submitted to CID experiments on an FTICR mass spectrometer. As a result, a total of 20 related substances (6 process-related substances and 14 degradation products) were identified. A degradation pathway was proposed and showed that most of the degradation impurities were obtained through oxidation. The possibility to acquire very informative spectra for thousands of compounds in few minutes make ultra-high-resolution mass spectrometers appropriate for the rapid profiling and comparison of different samples, which can constitute a real asset in the evaluation of pharmaceutical batches and impurities investigation. As in other application fields, semi-quantitative analysis, obtained through the comparison of peak areas, can highlight the presence of different impurities, which are due to discrepancies in drug manufacturing at various concentrations. Wu et al. [112] performed an LC-UV-ESI-FTICR comparison of four batches of methotrexate from 3 companies. In total, 15 impurities were identified. The relative abundance of 5 impurities exceeded 0.1% in one, two, or all batches. Then, direct infusion FTICR-MS^n^ was performed to (i) determine the fragmentation pathway of methotrexate, (ii) gather the MS^n^ fingerprints of 6 synthesized impurities and (ii) propose impurity structures using (i) and (ii). Complementary information on impurity structures was then obtained through UV spectra (chromophore information). The same principles of ultra-high-resolution mass spectrometry for impurity evaluation, applied for the analysis of small molecules, have been used with success in the field of protein-based drugs. For example, Wu et al. [113] performed an LC-Orbitrap (300,000 FWHM) analysis for the identification and quantification of arginine vasopressin impurities. Eight peptide impurities were identified: one isomer, three deamidation products, two amino acid deletion impurities, one amino acid insertion impurity, and one end-chain reaction product. The total mass fraction of all structurally related peptide impurities in arginine vasopressin was 30.3 mg/g ± 3.0 mg/g. In conclusion, FTICR and Orbitrap show advantages for drug formulation studies by giving access to information concerning both major compounds and impurities at very low concentrations, thanks to their sensitivity and the possibility to perform fragmentation experiments. Moreover, access to fine isotopic distributions allows confident molecular formula attributions and fast profiling and comparison. Nevertheless, the evaluation of drug formulations also includes compound distribution studies in the final pharmaceutical form, drug release, and interaction with the excipients of the formulation. When working with solution of the drug product for direct injection or chromatography, this information is not accessible as spatial information is lost, even using very high resolving power. In the next paragraph, we discuss the abilities of ultra-high-resolution mass spectrometry imaging as a powerful method to gain an additional layer of information for spatial characterization in drug formulation studies.

## 5. Ultra-High-Resolution Mass Imaging (MSI) in Pharmaceutical Research

One of the main challenges of research and development in the pharmaceutical industry is the investigation and comprehension of the biodistribution, metabolism, and accumulation of drugs in the human body. In fact, efficient drugs must reach target receptors at the site of action at a sufficiently high concentration, and at the same time, concentration should not be excessive to cause toxicity. Mass Spectrometry Imaging (MSI) has proved to be a powerful technique that enables the 2D (and more recently 3D) visualization of unlabeled molecule distributions within the surface of tissue sections or small clinical biopsies. Following the ‘4S-criteria for performance’, an ideal mass spectrometry imaging platform should satisfy four requirements, namely Spatial resolution, Sensitivity, Specificity, and high acquisition Speed. FT-based mass spectrometers have demonstrated good performances for imaging scopes, enabling the detection of a wide range of molecular weight compounds at ultra-high-resolution power (typically ~10^6^ at *m/z* 200) and mass accuracy (up to ppb). Nevertheless, as it happens for protein characterization, good performances in terms of spatial resolution, sensitivity, and specificity may require compromises in terms of acquisition speed. Of note, an increase in the applied magnetic field enables higher resolving power while keeping reduced transient lengths. Therefore, the use of high-field FTICR instruments (with the world’s highest being 21 T) helps in gaining more performant acquisition rates and mass accuracy over a long analysis time, in addition to reducing the pixel-to-pixel mass drift phenomena. As a proof of concept, improvements in mass resolution, accuracy, dynamic range, and spectral acquisition rates have been obtained in absorption-mode experiments through nano-spray desorption electrospray ionization (nano-DESI) coupled with a 21 T FTICR instrument. These findings stress the fact that both ion source technology development and applied magnetic field enable improvements in mass and spatial resolution for the imaging of small molecules. Even if high magnetic field instruments remain costly, MSI still results particularly important in pharmaceutical research for drug distribution and drug quantitation in a label-free approach.

Information on the pharmacodynamics/pharmacokinetics of small pharmaceutical molecules is usually obtained through time-resolved experiments in which sectioning is performed at different times after the local application of the drugs on the tissue. In the last years, this approach has been extensively developed for drug release and distribution studies in whole-eye segments [114,115], in the brain, or in skin sections [116]. As additional detailed examples, Handler et al. [117] compared MSI with dermal open-flow micro-perfusion (OFM), an in vivo minimally invasive probe technique for the quantification study of perfused drugs into the skin. The authors highlighted that even if MSI showed, in general, lower sensitivity compared to the OFM technique, it enabled direct mapping and quantification in customized skin regions, while OFM suffered from some limitations caused by difficulties in reaching tissue depth during implantation. In the same study, untargeted ultra-high-resolution MSI allowed to discriminate of drug molecules from skin components and to co-localize excipients in the same region as the drug, showing great potential in the discrimination of endogenous and exogenous metabolites in very complex samples. Besides the capabilities of the used analyzer, it must be taken into account that MSI outcomes are strictly dependent on sample preparation, especially in the pharmaceutical analysis of small molecules; relevant outcomes must be gained through the mastering of both tissue sectioning, sample treatment, and experimental setting. For example, a 54-fold improvement in MSI sensitivity for the distribution study of poorly ionized aminoglycosides antibiotics has been obtained through an accurate optimization of the washing time and matrix additive concentration, with respect to classic sample treatment coupled to a 7 T FTICR mass spectrometer [118]. Importantly, in comparison with classic high-resolution ToF analyzers, ultra-high-resolution mass spectrometers allow unequivocal chemical formula assignment due to their high mass accuracy and access to the isotopic fine structures. This may be of particular interest in the MSI distribution study of metal-based drugs or sulfur-containing drugs, which are nowadays used as anticancer, anti-rheumatics, antidiabetics, or antimicrobials. MALDI FTICR MSI has been used to distinguish isobaric oxaliplatin anticancer derivatives in human ovarian sections and study the selective localization of a new unknown Pt-isobar in the contour of the ovary, which was not discerned in previous studies based on the use of lower resolution analyzers (as the ToF) [119].

In addition to drug distribution studies, MSI based on ultra-high-resolution mass spectrometry has been extensively used in later years for the investigation of drug delivery systems. In fact, drug distribution and quantification in pharmaceutical delivery systems have been historically based on the use of fluorescent or radioisotopes probes labeling, leading to tedious sample preparation and targeted analysis, while ultra-high-resolution MSI constitutes a label-free approach. As recent examples, Zandanel et al. [120] applied MALDI-MSI to polycyanoacrylate nanoparticles loaded with doxorubicin, an anthracycline chemotherapy drug in mouse liver section. Based on a 7 T magnet analyzer, MALDI-MSI allowed the simultaneous detection and quantification of doxorubicin, polycyanoacrylate, and doxorubicinol, a metabolite of doxorubicin. The same approach has been used to follow the distribution of biodegradable coronary stents composed of lactic acid and glycolic acid in cross- and longitudinal sections of blood vessels [121]. More recently, MALDI-9T-FTICR has been used for the direct visualization of the drug release process from non-conductive polymeric-based long-acting parenteral implants (LAP) [122]. As to perform MSI, the surface must be conductive; the authors developed a platinum-coated sample surface treatment in combination with a metal sample holder, reaching MSI at spatial resolution of 50 μm, which resulted be comparable to other techniques for the study of LAPs, e.g., scanning electron microscope or laser ablation. Finally, another fast-growing field in the pharmaceutical analysis is represented by MSI based on ultra-high-resolution analyzers for drug distribution and drug metabolism study in 3D cell culture systems (i.e., spheroids and organoids). Palubeckaitė et al. [123] performed a proof-of-principle study in which the detection of doxorubicin was obtained by FTICR-MS on spheroids, whereas the drug was not visible by a Q-ToF instrument because of the presence of a close interfering peak. Overall, MSI based on ultra-high-resolution mass spectrometry suffers from some principal bottlenecks such as long analysis time and lack of structural information, especially for the MSI analysis of more complex molecules and biomolecules such as peptides and proteins. Chen et al. [124] integrated information obtained from MALDI-MSI (9.4T FTICR analyzer) and from droplet-based liquid micro-junction surface sampling liquid chromatography–high resolution mass spectrometry (LMJ-SSP-LC-HRMS) to study a proof-of-concept cyclic peptide, melanotan II. In the article, the authors showed that information on structures and intensities for melanotan II and its metabolites could be reached through LMJ-SSP-LC-HRMS while distribution and spatial resolution were reached through MALDI-FTICR-MSI at the expense of the acquisition time.

To go much further, with MSI providing 2D distribution of molecules within a tissue section, an extension of MSI to the 3D level (x, y, z axis, 3D-MSI) can provide more accurate information on drugs Pharmacokinetic/pharmacodynamics (PK/PD), as they are able to take into account heterogeneous organ characteristics and drug distribution. In fact, 3D-MSI gives access to bulk distribution information (surface and deep structure) of a tissue, opening the way for analysis of drugs In cells, organs, and ideally whole organisms, and 3D-MSI studies are therefore of utmost interest in the development of new drugs/therapies. Three-dimensional models are generally obtained by reconstruction of multiple layers imaged separately [125] or by performing deep profiling from several layers of mass spectral data from an entire sample. In complement, coupling of ion mobility (IM) separation with MSI (IM-MSI) performed at ultra-high-resolution capabilities may enable in-situ isobaric and isomeric separation according to their tridimensional conformation, increasing confidence in compound identification and spatial distribution mapping. Until now, most of the commercial platforms enabling IM-MSI are based on the use of ToF analyzers. Of note, both 3D-MSI and IM-MSI are very recent disciplines in which sample handling and analysis time are opposed to the need for fast responses in pharmaceutical development. In addition, dedicated platforms remain costly and need experienced analysts, circumscribing existing applications in the field of pure fundamental research [126,127]. Hopefully, the next years may be characterized by a true scientific revolution implying the development of new and accessible FT-based technologies for routine MSI analysis in the pharmaceutical industry.

### Quantitative MSI-FTICR

With the growing popularity of MSI, the need for quantitative MSI (qMSI) analysis has naturally increased in response to questions such as “where” and “how much” a drug is distributed in the target, preferentially in a single run analysis. This is especially relevant in toxicity and pharmacokinetic investigations but also in drug delivery profiling studies. However, the complexity of samples and the lack of sample clean-up or separation (for example, by chromatography) may represent a bottleneck in qMSI. In fact, drug ionization within the sample may change and suffer from interferences and matrix effects due to the presence of several ions. Thus, accurate quantification by MALDI-MSI still remains a challenge, and matrix effects or ionization efficiency should be assessed for a performant quantitative analysis based on imaging mass spectrometry. Quantitative MSI on ultra-high-resolution mass analyzers such as the FTICR has been developed following the same principle used for the optimization of quantitative MSI based on classic analyzers. In general, drug quantification in complex tissue sections is achieved through the construction of a calibration model obtained by spotting a dilution series of a reference standard onto the surface of a control sample [128]. Nonetheless, the uniform deposition of the standards results is difficult to achieve, and other methods, e.g., stable-isotope labeling of an analyte that is used as an internal standard, are preferred to perform a pixel-to-pixel correction and to consider ion suppression or matrix inhomogeneity effects [129,130,131]. Regarding recent pharmaceutical applications, Källback et al. [132] optimized a quantitative MALDI-MSI method based on an FTICR analyzer for mapping levels of the in vivo-administered drug citalopram, in mouse brain tissue sections, based on a combination of the two before mentioned methods. A dilution series of calibration standards, followed by a homogeneously applied stable isotopically labeled standard for normalization and a matrix on top of the tissue section, enabled the obtention of similar results to those from the reference method using LC–MS/MS —a golden standard for quantification objectives. In another study [133], the quantification of the pharmaceutical compound imatinib in gastrointestinal cancer tissues was obtained through a non-linear calibration curve that was constructed on a dilution series based on the computational evaluation of drug-containing areas of interest. This allowed us to obtain better data fitting and the assessment of the inherent method nonlinearities. In conclusion, it must be noted that even if efforts have been made in qMSI analysis (based or not on FT-based mass spectrometry), optimized methods for qMSI remain specific to the investigated system. Moreover, isotope labeled standards for every molecule may lack or be very costly. Appealing future perspectives point towards the development of a qMSI procedure based on the use of endogenous or exogenic markers for pixel-to-pixel normalization of MSI spectra.

## 6. Ultra-High Resolution for Untargeted Proteomics and Metabolomics

The development of medicine has been based on the characterization of the patient’s body response to a disease or drug treatment. Modern technologies allow us to perform this analysis at the molecular level, with the purpose of (i) obtaining insight into disease or drug mechanisms, (ii) monitoring health and disease development, and (iii) adapting the treatment as a result. The molecular response of an organism can involve genes, RNA transcripts, proteins, and metabolites. Studies concerning these different levels of molecular response are commonly known as “omics” sciences. Genomics and transcriptomics are commonly performed through sequencing and next-generation sequencing, representing complementary techniques not directly based on the use of mass spectrometry. On another side, MS-based proteomics and metabolomics represent nowadays a real gold standard for the evaluation of altered conditions (e.g., diseases) or to assess the effects induced by the introduction of exotic compounds in the body (e.g., pharmaceutically active compounds). Incomparably, the analysis of proteins and metabolites results is very challenging. Proteins undergo post-translational modifications (PTMs), presenting a higher complexity than the genome [134], while metabolites are characterized by a high degree of chemical diversity. In fact, the metabolome is made of millions of molecules with different functional groups, a wide range of polarity and pKa (e.g., amino acids, carbohydrates, lipids), structural isomerism, and chirality [135]. Proteomics and metabolomics include targeted (precursor selection) and untargeted (broadband acquisition) analysis. The latter creates large datasets with hundreds to thousands of features and often requires bioanalytical tools, e.g., statistical analyses, to determine metabolites or proteins distinctive of the disease studied. Among different MS platforms, ultra-high-resolution mass spectrometers can represent a real breakthrough in -omics studies, enabling highly precise molecular characterization of crude complex biological samples. In particular, the best performances of ultra-high-resolution mass spectrometry in -omics sciences have been reached by direct introduction (DI), methods that have become known as ‘‘shotgun’’ approaches (shotgun metabolomics, shotgun lipidomics, etc.). Shotgun omics approaches based on UHRMS lead to a drastic reduction of the acquisition time for each sample in opposition to coupling with chromatography. This is a discriminating factor for the choice of -omics methods because of the necessity to analyze very large datasets. Moreover, since proteins and metabolites of interest (e.g., disease markers) might be at low concentrations, FTICR and Orbitraps are instruments of choice for their unpaired sensitivity. Nevertheless, it is worth noting that -omics sciences are commonly performed using ESI as a preferential ionization technique. In this case, analysis of highly complex mixtures may suffer from matrix effects, especially for direct introduction analysis. This can introduce biases in the obtained -omics outputs and lead to an incomplete or erroneous understanding of the studied systems. Recently, direct introduction MALDI-FTICR MS has been used, beyond the MSI classic scope, to perform untargeted metabolomics showing reduced matrix effects with respect to DI-ESI-FTICR MS [136]. The use of alternative sources for ultra-high-resolution mass spectrometry in -omics may be envisaged and developed in the future. In health science and pharmaceutical research, metabolomics and proteomics have several applications in disease understanding, biomarker discovery, epidemiology, and toxicology. However, one should note that even with thousands of studies, a gap persists between discovery and clinical application. For example, many studies have been performed to study plant metabolism for active compound discovery, but those did not often lead to new drug development [137]. A compelling review by Muthu et al. [138] gives insights into reasons why disease-distinctive metabolite variations do not often translate into clinical targets, especially in the case of oncology.

In the case of pharmaceutical research, proteomics, and metabolomics studies have been performed to obtain a better insight into the organisms’ responses to diseases or treatments. For example, studies in laboratory conditions have been performed on LC-Orbitrap for glioblastoma multiforme in a murine model [139], the reaction of human neuroblastoma SH-SY5Y cells to manganese [140], and drug-induced lung injury on mice [141]. Regarding the last one, one should note that the authors demonstrated in a previous paper the advantage of a higher resolving power (240,000 FWHM) for lipid quantification [142]. Indeed, lipids are often present in organisms in mixes of very close chemical structures, which are not always separable using LC. Lipids often ionize with several adducts and exist with several unsaturation possibilities. For example, isobars such as [CE(16:0)+Na]^+^ (C_43_H_76_O_2_Na^+^, *m/z* 647.5737) and [CE(18:3)+H]^+^ (C_45_H_75_O_2_^+^, *m/z* 647.5761) would need a resolution of 250,000 FWHM to be clearly separated. Regarding biomarker discovery, the use of LC-Orbitrap in UHRMS has been described for esophageal squamous cell carcinoma [143] and hepatocellular carcinoma [144]. ESI-FTICR has been used for early-stage lung cancer [145], hypoxic ischemic encephalopathy [146], and Parkinson’s disease [147]. Other FTICR ionization sources were used for biomarker studies, such as electronic spray ionization for early gastric cancer biomarkers [148] and MALDI for Duchenne muscular dystrophy [149]. To conclude, it must be stressed that large-scale omics datasets (both from single- and multi-omics approaches) are difficult to handle and interpret manually. They require deep statistical analysis that allows for extracting distinctive features or biomarkers in an unbiased manner. For MS-based omics data, several approaches exist, ranging from classic multivariate analysis to more appealing predictive techniques based on machine learning and artificial intelligence. In addition, recent integrative multi-block approaches, namely DIABLO and MINT [150] within the mixOmics package [151], enable data integration from different omics platforms and levels. To go further, user-friendly web-based interfaces, e.g., XCMS [152], Workflow4Metabolomics [153], MetaboAnalyst [154], or MetaboDirect [155], allow MS-omics processing and statistical analysis for data derived from both Orbitrap and FTICR mass spectrometers.

### Analytical Developments

Comparison of “omics” results is challenging due to the difficulty of performing reproducible analyses. Indeed, many factors induce variation, from the type of sample and sample collection, sample storing, and acquisition method to the data analysis. A global effort has been performed in recent years by the “omics” community to standardize workflows. Mc Ardle et al. [156] suggested a standardized workflow for proteomics analysis of blood fluid using LC-ESI-Orbitrap and data-independent acquisition (DIA). DIA is a method used to fragment all precursors or within *m/z* ranges. DIA data analysis is performed using databases to correlate peptide fragments to proteins. The authors suggested two methods for high-throughput and mid-throughput LC-MS/MS. The chromatographic gradient length of the first method was 3-times shorter than the second. To maintain the global resolution of the method (including LC and MS), the MS^1^ resolution was higher for the high-throughput than the mid-throughput analysis, 120,000 FWHM and 60,000 FWHM, respectively. However, for MS^2^, the authors demonstrated that a lower resolution of 15,000 FWHM was required to increase fragment intensities for the best peptide pattern coverage. Subsequently, the study of protein expressions and metabolic responses to a disease or treatment requires a large number of patient samples (cohort with n > 100). Hence, fast and robust MS methods are required. Moreover, the sampling must be the less intrusive as possible for the patient’s well-being. Therefore, blood (essentially plasma) or urine is often used. Zhu et al. [157] developed a Flow Injection Analysis (FIA)-ESI-FTICR for the analysis of type 2 diabetes on mice that could detect and annotate approximately 1000 metabolic features within 5-min per sample. Regarding fast methods, Thompson et al. [158] developed an FIA-ESI- continuous accumulation of selected ions (CASI)-FTICR method for the metabolomic analysis of human plasma samples. CASI is an acquisition method that uses the quadrupole as a broadband mass filter to accumulate ions within a small mass range (e.g., 50 Da) in order to increase the dynamic range. Then, moving the filtered mass window across the entire *m/z* range allows the acquisition of the full spectrum. When compared to broadband acquisitions, CASI allowed better isotopic fine structure patterns, a signal improvement by five times, and a peak density increase of three times. Malinowska et al. [159] developed a spectral-stitching nano electrospray direct infusion (nESI-DIMS)-Orbitrap (120,000 FWHM) method using 96-well HTS platforms for the culture and extraction of HepaRG cells, which could be used for toxicological testing and therefore was faster than usual toxicological studies. Wang et al. [160] developed an LC-ESI-Orbitrap (120,000 FWHM) data-independent acquisition workflow for single-cell proteomics that could identify up to 1500 protein groups using only 0.2 ng of sample and a 15-min LC-gradient. Bayne et al. [161] developed a polarity switching FIA-ESI-FTICR method for metabolomics that allowed the detection of metabolites from 11 metabolic classes (from carbohydrates to lipids) in only 5 min. Direct tissue analysis, using ionization sources such as the internal extractive electrospray ionization (iEESI) source, can lead to faster analysis due to the withdrawal of the sample extraction. In iEESI, the extraction solution (e.g., methanol, water) charged at a high voltage (±4.5 kV) is directly infused into the solid sample (e.g., a tissue) through the inserted capillary, allowing extraction and ionization in a single step [162]. As an example, iEESI-Orbitrap was used for biomarkers discovery related to the progress of colorectal cancer [163]. The use of the iEESI source has recently been reviewed and showed low limit of detection (0.002–0.100 µg/L) for a variety of pharmaceutical compounds [164].

Finally, instrumental development can utilize other separation techniques, such as ion mobility, for a higher metabolite/ protein coverage. High-field asymmetric-waveform ion-mobility spectrometry (FAIMS) is a technology that can be implemented on Orbitrap mass spectrometers after the ion source. In FAIMS, ions are transmitted within an asymmetric waveform (alternating high and low electric fields) based on their difference in ion mobility in the gas phase. The addition of a compensation voltage (CV) allows the selection of a group of ions with precise mobility. Rapid CV change can allow overlapping peptides deconvolution and contaminant removal. Hebert et al. [165] compared human cell line proteomics analyses using different Orbitrap (420,000 FWHM for qualitative analysis, 120,000 FWHM for quantitative analysis) and experiments: direct infusion-ESI-MS, LC2D-ESI-MS, and direct infusion-ESI-FAIMS-MS. The authors demonstrated that the use of FAIMS in a direct-introduction experiment could boost peptide identifications by up to 2-fold and protein identifications by up to 55%. Moreover, when compared to LC2D-MS, direct introduction-FAIMS could generate a close number of protein identifications but with a much simpler experimental set-up.

## 7. Ultra-High Resolution for Environmental Analysis

The ever-growing use of medication globally, and the inefficient removal of numerous pharmaceuticals by traditional wastewater treatment systems, have caused a substantial rise of pharmaceuticals in the environment [166]. Moreover, human metabolization and transformation in the environment (e.g., oxidation, photolysis, biotransformation) of pharmaceuticals lead to the generation of transformation products (TPs) that can be pharmaceutically active, hence potentially toxic to the environment. Pharmaceutical pollution in the environment can be studied using target and/or suspect screening or untargeted mass spectrometry analysis. Target analysis consists of the absolute quantification of pharmaceuticals using reference standards and is often performed using low-resolution instruments, e.g., triple quadrupole. Since environmental samples are varied and complex matrices (e.g., water, soil, air), and pharmaceuticals are often in low concentration, selective sample preparation is required for the removal of matrix interferences. Although this methodology can perform unambiguous identifications and quantifications, it is limited by the availability of standards and prior knowledge of the sample. The identification of pharmaceutical transformation is currently a work in progress; hence, target analyses often exclude them due to a lack of standards. Therefore, suspect screening was developed as a close methodology that relies on databases (e.g., molecular formula, *m/z*, MS² fragments) of expected (“suspected”) compounds. In this case, ultra-high-resolution mass spectrometers are preferred for the acquisition of the broadband spectrum (untargeted acquisition), followed by identification using databases. This allows the potential to identify hundreds of chemicals in a single run without standards [167]. Untargeted analysis of environmental samples takes the most profit from the ultra-high-resolution mass spectrometer capabilities as these samples are complex matrices made of thousands of chemicals. Moreover, untargeted analysis can be performed with a minimum sample preparation, which limits the loss of important information. FTICR has been extensively used for the characterization at the molecular level of dissolved organic matter (DOM), a highly complex mixture of organic molecules (mostly < 1000 Da) that plays an important role in regulating biogeochemical processes. DOM has gained interest in recent years, especially for the characterization of the impact of human activity (e.g., pharmaceutical contamination) on the environment [168]. A particular methodology was developed for the analysis of complex matrices, such as DOM, by UHRMS. Thanks to the high mass accuracy of the FTICR or the Orbitrap, thousands of molecular formulae can be automatically assigned using the appropriate limitations (e.g., ^12^C (1–60), ^1^H (1–120), ^14^N (0–3), ^16^O (0–30) and ^32^S (0–1), 0.5 ≤ H/C ≤ 3 and O/C ≤ 1.5) [169]. Then, further analyses are proceeded using graphical representations, e.g., the Kendrick diagram, a double bond equivalent vs. carbon number plot, and the van Krevelen diagram [168,170]. Although most DOM studies are performed by FTICR, Hawkes et al. [171] showed that Orbitrap at a resolution of 100,000 FWHM was also an appropriate technology for DOM analysis, especially for very low masses (50 ≤ *m/z* ≤ 100). However, the authors noted limitations in the resolution of isotopic distributions. For example, the isotopes [CHO]C_3_ and [CHO]H_4_S (Δ_m_ = 3.34 mDa), which are used for the confident identification of sulfur compounds, could not be resolved. Herein, a few examples of pharmaceutical contaminations in various matrices, their impact on the environment, and technological developments for their removal will be presented. DOM analyses will especially highlight the advantage of UHRMS for the analysis of complex samples composed of thousands of signals.

### 7.1. Pollution in the Environment

Due to the ever-growing use of pharmaceuticals and the real struggle to efficiently remove them from the environment, humans, among other species, are involuntarily exposed to plentiful pharmaceuticals and their transformation products. Pharmaceuticals are converted by human enzymes to phase I and phase II metabolites; thus, target screening of pharmaceuticals might not show exposure to them. Liu et al. [172] developed an LC-Orbitrap-MS/MS (120,000 FWHM) method and database for the analysis of the enzymatic digestion products from 139 xenobiotics (environmental contaminants, pharmaceuticals, and dietary/personal care products) that could give insight into the pharmaceutical use of a healthy cohort (n = 120) with undocumented xenobiotic exposure. Although this methodology is promising, this is a work in progress since the database needs to be completed for the approximately 3000 compounds that are estimated to be used as pharmaceuticals [173]. Target and suspect screening has been used for various environmental matrices and pharmaceuticals, e.g., antibiotics in water ponds (LC-Orbitrap 140,000 FWHM) [174], antibiotics in groundwater and their interaction with DOM (LC-Orbitrap 100,000 FWHM) [175], and emerging contaminants extracted from Antarctic phytoplankton (ESI-FTICR) [176]. These studies showed that even after sample cleaning, the matrix was still complex, justifying the use of ultra-high resolution for the confident discrimination of coeluting and isobaric compounds. Moreover, these few examples demonstrate that pharmaceuticals are ubiquitous in the environment, e.g., 42 pharmaceuticals identified in the Antarctic phytoplanktons study. Thanks to the use of wastewater treatment plants (removal efficiency of approximately 90 %), pharmaceutical pollution is limited. However, as is presented in the next part, the complete removal of pharmaceuticals and the production of non-toxic wastewater are laborious.

### 7.2. Wastewater Treatment Plant (WWTP) Effluents

In WWTPs, water effluents are usually treated by the combination of several processes: coagulation/flocculation, biological treatment, advanced filtration, advanced oxidation, or hybrid technologies [177,178]. The efficiency of WWTP is followed by several global environmental parameters, e.g., biological oxygen demand and chemical oxygen demand. Even though these parameters usually show good removal efficiencies of organic matter in WWTPs (≥90%), information that can be obtained on molecular composition is limited. Actually, numerous studies show that most common WWTP treatments are inefficient in removing pharmaceuticals and lead to the formation of transformation products (TPs) [179]. Active pharmaceutical Ingredients (API) and some TPs are usually monitored in wastewater using target analysis with triple quadrupoles, which is limited by the number of compounds analyzed per acquisition and cannot perform the analysis of hundreds to thousands of contaminants—unless by tremendous method development efforts. On the other hand, untargeted screening consists of the acquisition of the full MS spectra and, with ultra-high resolution, can be used for the analysis of thousands of compounds. Yet, due to the diverse molecular characteristics of pharmaceuticals, the interpretation of pharmaceutically polluted environmental sample spectra is excessively challenging. Therefore, some untargeted studies rely on the use of target and suspected databases for identification. For example, Perkons et al. [41] developed a strategy for the target and suspect screening of hundreds of pharmaceuticals and their transformation products (>500) from the inlet and outlet of WWTPs using direct infusion-ESI-FTICR. Identification was performed using an experimental MS² database completed by predicted fragmentations and calculation of the theoretical *m/z* of fragments. The authors noted that this strategy allowed a better matching between the database and FTICR MS² spectra since most experimental MS² spectra are collected using mass spectrometers with a less accurate mass measure than FTICR. From the 58 active pharmaceutical ingredients (APIs) and 18 TPs found in the inlet, 38 APIs and 16 TPs were found in the outlet, illustrating the difficulty of efficiently removing pharmaceuticals using WWTPs. Brunner et al. [180] developed a strategy that integrates data from targeted and untargeted analyses by LC-ESI-Orbitrap (120,000 FWHM) and bioassays for the assessment of two WWTP pilots. The untargeted analysis resulted in 927 and 310 features in positive and negative ionization, respectively. Even after using an extensive database of 9560 suspects, over 1000 features remained unidentified, demonstrating the need for analyses complementary to database search. Combining bioassay results with the hierarchical analysis of untargeted data allowed us to determine 24 and 27 potentially harmful transformation products. Even though these features were not identified due to poor MS² and no match in databases, these results highlight that toxic compounds from the effluents of WWTP might be unknown molecules. Hence, there is a need for a better characterization of TPs, especially knowing that some TPs are more harmful than their parent compound have been described [179].

In most WWTP, degradation of organic contaminants is performed using biological processes (e.g., activated sludge, membrane bioreactors, moving bed bioreactors, and constructed wetlands) relying on additional organic matter as a primary growth substrate [179]. The composition of DOM in wastewater is hardly described, although it can act as a precursor of disinfection by-products and transporter of pollutants and affect the performance of WWTPs [42]. This is possibly due to the molecular complexity of DOM (thousands of molecules), which requires UHRMS for complete characterization, while most environmental studies are performed using low-resolution to high-resolution mass spectrometers. Most DOM analyses are performed using ESI-FTICR, as in the following examples. Using DOM determination and the quantitative monitoring of the degradation of pharmaceuticals in a WWTP pilote, Hellauer et al. [181] showed that the DOM composition was correlated with the degradation of pharmaceuticals. In their analysis of DOM from the WWTP of a hospital, Gan et al. [42] showed that the molecular diversity of DOM varied alongside the treatment and increased after the WWTP treatment (from 2927 to 3217 chemical formulae) even though the total nitrogen content and dissolved organic carbon content were reduced. By studying the transformation of DOM in a pharmaceutical wastewater effluent during ozonation WWTP treatment, Shi et al. [182] showed that ozone dosage had an influence on the DOM composition. Hu et al. [183] and Liao et al. [184] showed that the formation potential of N-nitrosodimethylamine, a highly controlled carcinogenic disinfection by-product, was correlated with the presence of nitrogen-containing DOM with a mass weight between 3 kDa and 10 kDa (e.g., peptides, small proteins). Still, Hu et al. [183] observed that the treatments that followed in the WWTP (aerobic sludge and moving bed biofilm reactor) were effective at removing N-nitrosodimethylamine-precursors, demonstrating that pharmaceutical wastewater treatment requires a combination of various processes. In these examples from WWTP studies, we observed that molecular formula characterization gave access to a better understanding of wastewater treatments. Additionally, ultra-high resolution was required to identify these hundreds to thousands of chemical formulae.

### 7.3. Novel Technologies for (Bio)Remediation

The examples presented in the last part showed that pharmaceutical removal is highly challenging. Hence, new technologies are being developed, and associated transformation products need to be identified. Orbitrap and FTICR are instruments of choice for TP characterization since these compounds are at low concentrations in complex mixtures. Moreover, the obtention of highly mass-accurate fragments is beneficial for structural analysis. Advanced oxidation processes are promising technologies to efficiently remove a wide range of recalcitrant pollutants by reaction with generated strong radical oxidants (mostly hydroxyl radical HO^•^ and sulfate radical SO_4_^•−^). For example, removal rates and transformation product studies performed by LC-ESI-FTICR showed promising results for the treatment of ibuprofen [185] and maprotiline [186]. Studies of other novel treatments by ESI-FTICR also include carbon nanotubes enriched in heteroatoms catalysts with ozone [187] and the use of the white fungus *Trichoderma harzianum* and *Pleurotus ostreatus* [188].

As a concluding remark for this part on the analysis of pharmaceuticals in the environment, we comprehended how UHRMS could be used for the analysis of complex matrices and/or traces in a complex matrix. UHRMS shows unbeatable performances for the assignment of thousands of molecular formulae in complex samples, especially for DOM. WWTP effluent’s molecular characterization is a challenging work in progress, and thrilling new studies will surely come out in the future. It would be interesting to see more studies on the characterization of pharmaceuticals and their transformation products in DOM. One particularly useful tool for these analyses would be molecular networking, a tool based on MS² data that links compounds with similar structures and provides “molecular networks” as a visualization. As an example, Petras et al. [189] showed that DOM could be analyzed using molecular networking from LC-Orbitrap-MS/MS (140,000 FWHM) data. Then, combining qualitative and quantitative data from different techniques might give the most information for environmental analysis.

## 8. Conclusions and Perspectives

The use of ultra-high-resolution mass spectrometry for pharmaceutical analysis and its advantages over high-resolution mass spectrometry were overviewed. FTICR and Orbitrap instruments offer compelling features that can allow the molecular characterization of complex samples and often much faster analyses than other analytical techniques, e.g., direct introduction FTICR compared to LC-HRMS. Their great sensitivity and dynamic range, especially for the FTICR, allow trace analysis, e.g., impurities in the formulation and environmental analyses. The possibility to perform diversified activation methods, such as IRMPD, ECD, and ETD and their high mass accuracy empower structural analyses. This is essential for drug discovery, drug metabolite, and transformation product identification. On another side, ultra-high resolution guarantees to go further in chemical characterization thanks to (i) the access to the fine isotopic distribution of ions, which, combined with high mass accuracy, provides confidence in molecular formula annotation, (ii) the increase in peak capacity and (iii) isobaric separation. Therefore, when compared to HRMS, UHRMS clearly enhances the analysis of complex matrices of thousands of signals, e.g., dissolved organic matter in environmental studies or tissues for drug and its metabolite biodistribution studies. Although there is a tremendous number of papers using Orbitrap for pharmaceutical analysis, most of them were performed with resolution settings under 100,000 FWHM. As shown in this review, ultra-high-resolution pharmaceutical analysis is, for the moment, mostly centered on the use of the FTICR, as demonstrated by a higher number of cited papers using this technique. With the new generation of Orbitraps, which allows a higher resolution for the same acquisition frequency, the number of studies based on the use of Orbitrap instruments should increase in the future. Similarly, the new generation time-of-flight platforms, able to reach an ultra-high resolution, may represent a user-friendly and less expensive alternative, foretelling a higher use of UHRMS in pharmaceutical analyses within the next years.

In perspective, improvements in instrumentation, such as better data acquisition rate, hyphenation with mono- and multi-dimensional chromatography techniques compatible with ultra-high resolution, and the integration of flexible and intuitive software for data analysis, are still highly demanded.

From another point of view, it is highly desirable to extend the use of UHRMS in pharmaceutical analysis beyond research scopes, which underlies the integration of such techniques in the routine practice of the pharmaceutical industry. To this extent, the scientific community should put effort into standardizing protocols and delivering robust and universal methods for pharmaceutical analysis based on the use of UHRMS instruments. To go much further, while more accessible and less costly instrumentations are not easy to obtain, it is auspicious to encourage researchers to share their knowledge and establish collaborations with the scientists working in the pharmaceutical industry for a more conscious and efficient utilization of these cutting-edge techniques at the service of human health.

## Figures and Tables

**Table 1:** Global comparison of Orbitrap and FTICR instruments. Information was retrieved from [6].

	Orbitrap	FTICR
Characteristics	Expensive	Very expensive
	Compact instrument	Heavy and massive instrument
	High electric field in the analyzer	High magnetic field requiring cryogenic maintenance
User experience	User-friendly	High expertise required
	Numerous parameters automatically set	Fine-tuning of parameters required—high influence on resolution
Mass accuracy (ppm)	0.5–5	0.05–1
Resolution (FWHM)	120,000–1,000,000	200,000–10,000,000
Coupling to LC/GC	Easy	Difficult
	Instrument sold with setup	Requires parameters optimization
Distinctive features	Automatic gain control (AGC) and normalized level (NL)	Electrospray Ionization (ESI)/ Matrix-Assisted Laser Desorption Ionization (MALDI) dual source
	eFT	Absorption mode
	Higher-energy collisional dissociation (HCD)	2D-FTICR
	Electron transfer-HCD (ETHcD)	Electron detachment dissociation (EDD)
Limitations	Space-charge effects, especially for compact Orbitrap	High magnetic field magnets difficult to produce
	Requires high voltages (4–5 kV) -> technical difficulty to avoid power supply variation	On current commercial FTICR: no automatic adjustment of the number of ions entering the cell (i.e., AGC)

## Data Availability

The data presented in this study are available on request from the corresponding author.
